# Cold Saline Perfusion before Ischemia-Reperfusion Is Harmful to the Kidney and Is Associated with the Loss of Ezrin, a Cytoskeletal Protein, in Rats

**DOI:** 10.3390/biomedicines9010030

**Published:** 2021-01-03

**Authors:** Csaba Révész, Anita A. Wasik, Mária Godó, Pál Tod, Sanna Lehtonen, Gábor Szénási, Péter Hamar

**Affiliations:** 1 Institute of Translational Medicine, Faculty of Medicine, Semmelweis University, 1143 Budapest, Hungary; revcsab@gmail.com (C.R.); godmar@net.sote.hu (M.G.); todpal90@gmail.com (P.T.); szenasi.gabor@med.semmelweis-univ.hu (G.S.); 2 Department of Pathology, Faculty of Medicine, University of Helsinki, FIN-00014 Helsinki, Finland; anita.wasik@helsinki.fi (A.A.W.); sanna.h.lehtonen@helsinki.fi (S.L.); 3 Research Program for Clinical and Molecular Metabolism, University of Helsinki, FIN-00014 Helsinki, Finland

**Keywords:** organ preservation, acute kidney injury, cold perfusion, cytoskeleton, ezrin

## Abstract

Background: Organ protection for transplantation is perfusion with ice-cold preservation solutions, although saline is also used in animal experiments and living donor transplantations. However, ice-cold perfusion can contribute to initial graft injury. Our aim was to test if cytoskeletal damage of parenchymal cells is caused by saline itself or by the ice-cold solution. Methods: F344 rat kidneys were flushed with cold (4 °C) saline, ischemic and sham kidneys were not perfused. In a separate set, F344 kidneys were flushed with saline or preservation solution at 4 or 15 °C. Ischemia time was 30 min. Results: Renal injury was significantly more severe following cold ischemia (CI) than after ischemia-reperfusion without flushing (ischemia/reperfusion (I/R)). Functional and morphologic damage was accompanied by severe loss of ezrin from glomerular and tubular epithelial cells after CI. Moreover, saline caused serious injury independently from its temperature, while the perfusion solution was more beneficial, especially at 4 °C. Conclusions: Flushing the kidney with ice-cold saline can cause more severe injury than ischemia-reperfusion at body temperature even during a short (30 min) ischemia. Saline perfusion can prolong recovery from ischemia in kidney transplantation, which can be prevented by using preservation solutions.

## 1. Introduction

Organ preservation historically focuses on the reduction in anoxic injury by cooling organs to ~4 °C [1]. However, severe hypothermia has well known deleterious effects especially if combined with anoxia [2]. The cold induces structural changes to cell membrane lipid bilayers and hypoxia induces Na^+^/K^+^ pump dysfunction, leading to edema [3,4]. Cold storage-induced injury is a pivotal contributing factor to early graft dysfunction [5]. Prolonged cold ischemia causes primary non-function or dysfunction of the transplant [1]. Extreme cold (<5 °C) storage can be deleterious to anoxic organs as demonstrated in the case of amputated limbs. The deleterious effects of cooling were reduced by intermediate (10 ± 5 °C) hypothermia of the tissue [6]. Presently, preservation solutions are used to reduce cold ischemic injury of organs stored for hours. However, if the storage period is short, such as in the case of living donations [1,7] or rodent models of organ transplantation [8,9,10,11,12], simple cold saline is often used to flush the blood from the donor organ. This approach is based on the generally held view that living donations do not require long-term organ storage so the application of complex storage solutions is less important [1]. However, even a short anoxic storage following cold saline perfusion may be harmful to the kidney.

Preservation injuries have already been documented in 1976 [13] and cooling plays a role in their development [14]. Although oxygenated ex-vivo normothermic perfusion (EVNP) has been used before [15], currently, the very simple static cold storage is the generally applied form of organ preservation since its introduction by Collins [16] in 1969. EVNP has recently been suggested to be a better form of organ preservation than cold storage due to the lack of tissue damage [17,18]. Cells may respond to the cold by cytoskeletal rearrangement or disruption [14,19,20,21,22]. Ezrin, a cytoskeletal linker protein, plays important roles in both podocyte [23,24] and tubular epithelial cell (TEC) [25,26,27] functions. Thus, investigating optimal organ storage for transplantation is a current issue.

To investigate if flushing with 4 °C cold saline protects from or aggravates ischemic acute kidney injury, we investigated Fischer rat kidneys subjected to 30 min ischemia with or without flushing with 4 °C cold saline or preservation solution at low (4 °C) or intermediate (15 °C) temperatures.

## 2. Materials and Methods

### 2.1. Surgical Procedures

All protocols were approved by the Pest County Government Office and the Animal Ethics Committee of Semmelweis University (PE/EA/2202-5/2017). All experiments were performed in accordance with the Hungarian Acts XXVIII of 1998 and LXXVIII of 2018 on the protection and welfare of animals and EU Directive 2010/63/EU for animal experiments.

Eight-week-old inbred, male, Fischer (F344: F, (RT11v1)) rats were used in the experiments. Rats were randomized into each study group based on their body weight, so that initial bodyweight was similar in all groups. Average bodyweight was 258 ± 35.5 g at the start of the study and did not change significantly throughout the observation period. The number of rats was 8 in all groups.

All surgical procedures were performed under pentobarbital (Nembutal, Ceva-Phylaxia, Budapest, Hungary) anesthesia on preheated operating tables with constant monitoring of the rectal temperature (Physiological-Biological Temperature Controller TMP-5b, Supertech Ltd., Pécs, Hungary). The rectal temperature of the rats was 37.0 ± 0.3 °C. The left kidney was isolated from its surroundings by decapsulation. The renal artery and vein were isolated and occluded with atraumatic clamps (Aesculap, BBraun, Budapest, Hungary, FD562) for 30 min. The total ischemia time was set to 30 min in all groups. To investigate the role of perfusion with ice cold saline following occlusion of the renal artery and vein, a small incision was made on both the renal artery and vein. The renal artery was cannulated with a 24 G pediatric intravenous catheter and was flushed with a 10 mL syringe filled with 4 °C saline (ice cold saline flushed cold ischemic group: CI). In a separate experiment, kidneys were flushed with saline or Custodiol (Dr. Franz Köhler Chemia GMBH, Bensheim, Germany) at 4 or 15 °C. The perfusion was considered complete when all the blood was removed from the kidney according to visual control. Next, the blood vessel incisions were closed with a micro suture (10.0 atraumatic prolene). As a comparison for the ischemic injury, in-situ ischemia/reperfusion (I/R) without flushing with cold saline was performed. In order to investigate the extent of renal function loss due to a right nephrectomy and to control for a possible influence of renal decapsulation and blood vessel preparation, a sham group (sham) was included. The sham operation included decapsulation and isolation of the renal artery and vein of the left kidney. The right kidney was removed in all groups, following the left kidney operation during the same operative session, and was preserved for control purposes.

### 2.2. Morphologic and Molecular Methods

In order to investigate the morphologic and molecular effects of ischemia with or without cold flushing, 1, 3, and 5 days after surgery, animals were anesthetized, blood was removed from the circulation by trans-aortic perfusion with 4 °C saline and the left kidneys were removed, cut with a sterile scalpel-blade and fixed in formalin or liquid nitrogen.

Renal retention of creatinine and urea in the blood was measured from 32 μL whole blood on a Reflotron IV automated analyzer (Boehringer Mannheim, Germany) with a fast-test-stripe as described previously [28,29]. All analytical procedures were performed in duplicate.

### 2.3. Morphology

The lower poles of the left kidneys were immersion fixed in 4% phosphate buffered paraformaldehyde for 24 h followed by dehydration and embedding in paraffin for morphological analysis. Four µm thick sections were deparaffinized, rehydrated and stained with hematoxylin–eosin (HE) or periodic-acid–Schiff (PAS). Samples were examined in a blinded fashion. Representative sections of the cortico-medullary region were photographed (Leica DC500, Leica, Wetzlar, Germany) including a few paramedullary glomeruli.

Quantitation of renal damage included the evaluation of inflammatory cell infiltration of the kidney, and the ischemic tubular damage was estimated using a previously published scoring system [30]. Different levels of tubular damage (nuclear atypia, TEC vacuolization, reduction in TEC height leading to tubular luminal dilation and TEC detachment or hyalinization) were defined. PAS-stained kidney samples were scored at ×200 absolute magnification, and a score from 0 to 3 was given for each tubular profile per field of view: 0 = normal histology; 1 = tubular cell swelling, brush border damage, vacuolization of TECs; 2 = moderately dilated tubules, more severe brush border loss, edematous TECs, focally weak/lost nuclear staining; 3 = total tubular necrosis, no nuclear staining, and dilated tubules. Mean values were calculated from 10 fields of view.

### 2.4. Immunoblots and Indirect Immunofluorescence

Western blotting was performed with mouse anti-ezrin (clone 3C12) [31] (Abcam, Cambridge, UK), mouse anti-actin and mouse anti-protein disulfide isomerase (PDI) (Sigma-Aldrich, St. Louis, MO, USA) IgGs in duplicate as previously described [32,33]. Blots were quantified using an Odyssey Infrared Imaging System (LI-COR, Lincoln, NE). Rat kidney cryosections were fixed with acetone and stained with mouse anti-ezrin IgG as described [34] followed by detection with AlexaFluor 488 donkey anti-mouse IgG (Molecular Probes, Life Technologies, Carlsbad, CA, USA). The samples were examined with a Zeiss Axioplan2 microscope (Carl Zeiss Microscopy GmbH, Jena, Germany) and photographed using the same settings for each sample. Photographed samples were scored and a score from 0 to 4 was given for each glomerulus and tubulointerstitial field of view separately: 4 = intense and sharp staining in >75% of the glomeruli or tubules, 3 = slightly reduced staining in <50% of the glomeruli or tubules, 2 = reduced staining in 50–75% of the glomeruli or tubules, 1 = faint staining of >75% of the glomeruli or tubules, 0 = practically no staining, only faint contours of glomeruli or tubules. Mean values were calculated from 5 fields of view.

### 2.5. Statistical Analysis

Results are presented as mean ± standard deviation (SD). Continuous variables were compared using either one-way analysis of variance (ANOVA), followed by the Dunnett’s multiple comparison post hoc test versus the sham group, or two-way ANOVA with Tukey’s multiple comparisons test or Kruskall–Wallis test (Microsoft Excel, Microsoft Corporation, Redmond, Washington) if variances differed significantly based on Bartlett’s test. Linear correlation was assessed with Pearson product-moment correlation coefficient. The null-hypothesis was rejected if the *p* value reached statistical significance (*: *p* < 0.05).

## 3. Results

### 3.1. Renal Function

**Figure 1:**
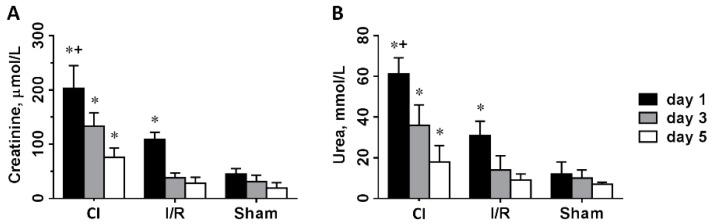
Glomerular and tubular functional parameters, creatinine (**A**) and urea (**B**) retention 1, 3 and 5 days after left kidney operation. CI: cold ischemia, I/R: ischemia-reperfusion, Sham: sham operated (n = 8 in all groups). *: *p* < 0.05 vs. sham, +: *p* < 0.05 vs. I/R (day 1).

Creatinine (Figure 1A) and urea (Figure 1B) retention were most severe on day 1 and remained elevated on day 3 and 5 in ischemic kidneys flushed with cold saline. We observed significant retention of creatinine and urea on the first postoperative day in I/R animals, which diminished on days 3 and 5. Sham-operated animals had normal kidney function during the observation period. Creatinine and urea levels were already within the reference range one day after operation, but there was a tendency of creatinine and urea elimination on days 3 and 5. Thus, acute kidney failure was more severe and lasted longer in kidneys flushed with cold saline vs. similar ischemia duration when kidneys were buffered with normal temperature own blood.

### 3.2. Morphologic Analysis of Kidneys

**Figure 2:**
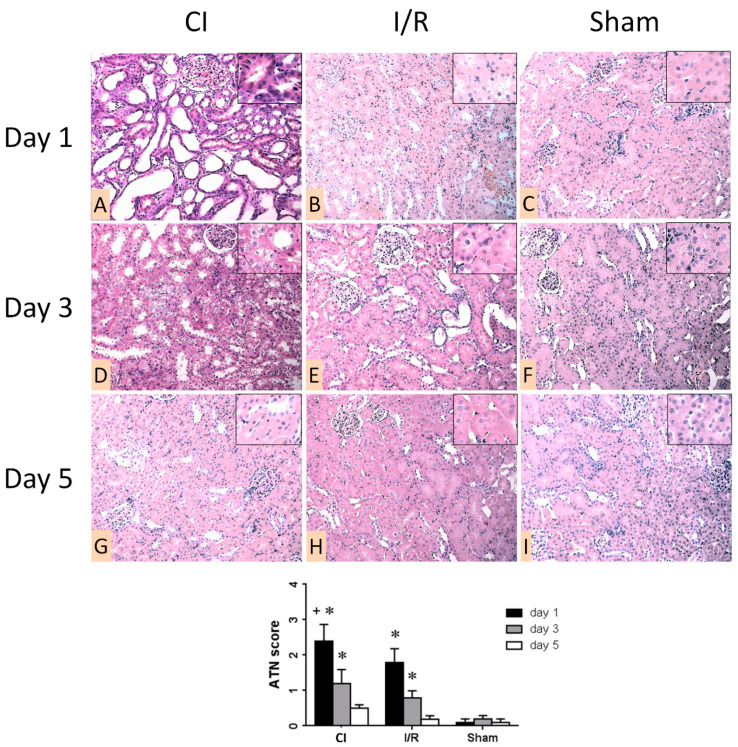
Representative morphologic images of operated left kidneys one (**A**–**C**), three (**D**–**F**), and five (**G**–**I**) days after the operation. First column (**A**,**D**,**G**): cold ischemia (CI). Second column (**B**,**E**,**H**): ischemia-reperfusion (I/R). Third column (**C**,**F**,**I**): sham. (hematoxylin–eosin (HE) staining, 200×). Inserts show proximal tubular damage (1000×). Graph: quantitative evaluation of acute tubular necrosis (ATN) score 1, 3 and 5 days after the operation (n = 8 in all groups). *: *p* < 0.01 vs. sham, +: *p* < 0.05 vs. I/R (day 1). (HE staining, 200×).

Sham kidneys were without apparent morphologic alterations at any time-point (Figure 2C,F,I). At day 1, acute tubular necrosis (ATN) was obvious in A and I/R kidneys, and it was more severe in A than in I/R (Figure 2A,B). ATN diminished on day 3 (Figure 2E) and disappeared on day 5 in I/R (Figure 2H). The disappearance of ATN signs was slower in A compared to I/R (Figure 2G,H). ATN score was higher in A than in I/R kidneys on days 3 and 5 (Figure 2/graph).

### 3.3. Western Blotting and Immunostaining for Ezrin

**Figure 3:**
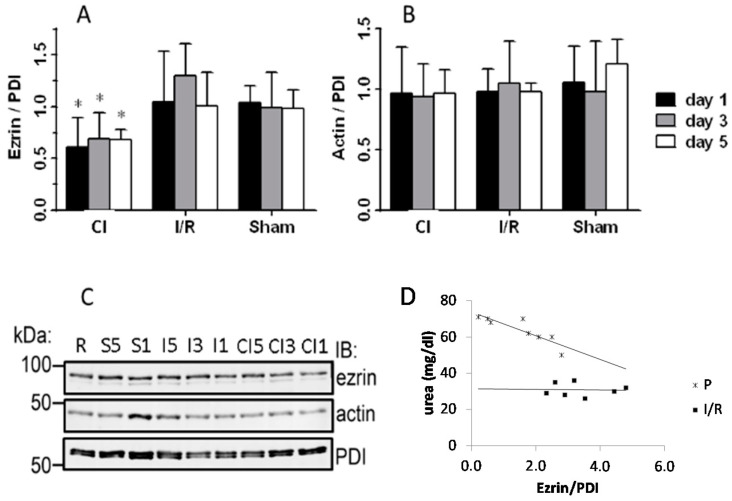
Ezrin and actin expression determined by Western blotting. Western blot image of ezrin, actin and protein disulfide isomerase (PDI) expression. Representative image from nine independent measurements. The samples are: non-ischemic right kidney (R), sham (S5, S1), ischemia-reperfused (I5, I3, I1) kidneys and cold-saline flushed ischemic kidneys (CI5, CI3, CI1). Quantification of Western blots (**B**,**C**) 1, 3 and 5 days after the operation. The average control value measured in the right kidneys was set to 1. Different blots were compared after normalization to a sample included in all 9 blots (n = 8 in all groups). (**A**) Quantification of ezrin presented as ezrin/PDI ratio. *: *p* < 0.05 vs. corresponding sham. (**B**) Quantification of actin presented as actin/PDI ratio. Actin expression was not influenced significantly. (**C**) Representative Western blots of ezrin, actin and PDI at various days after the interventions. (**D**) Correlation of ezrin content with blood urea level. The data shown were obtained 1 day after the operation in rats with cold-saline flushed ischemic kidneys (y = 6.539x + 73.741, R2 = 0.7644, *p* < 0.01) or in ischemia-reperfused kidneys (y = −0.1755x + 31.453, R2 = 0.00196, NS = not significant); the slopes are significantly different (*p* < 0.01).

In cold-saline flushed ischemic kidneys the ezrin/PDI ratio was reduced to 61%, 69% and 68% of the right kidney 1, 3 and 5 days after ischemia, respectively (Figure 3C). Thus, flushing with cold saline combined with ischemia caused a severe and prolonged reduction in ezrin in cold-saline flushed ischemic kidneys that was not observed after ischemia alone.

The expression level of ezrin normalized to PDI (Figure 3A) was similar in the I/R and sham groups compared to the average ezrin/PDI ratio measured in the right kidneys.

As the proteins generally used for the normalization of Western blots, actin [35] and tubulin, are both parts of the cytoskeletal network and may be disrupted by cold temperature, we used PDI, an enzyme of the endoplasmic reticulum, which is also commonly used as a loading control [36]. Actin expression, normalized to PDI, was not significantly influenced by ischemia with or without flushing with cold saline (Figure 3B).

Expression of ezrin correlated with urea retention only in animals one day after the operation. No such correlation was observed in I/R animals (Figure 3D).

**Figure 4:**
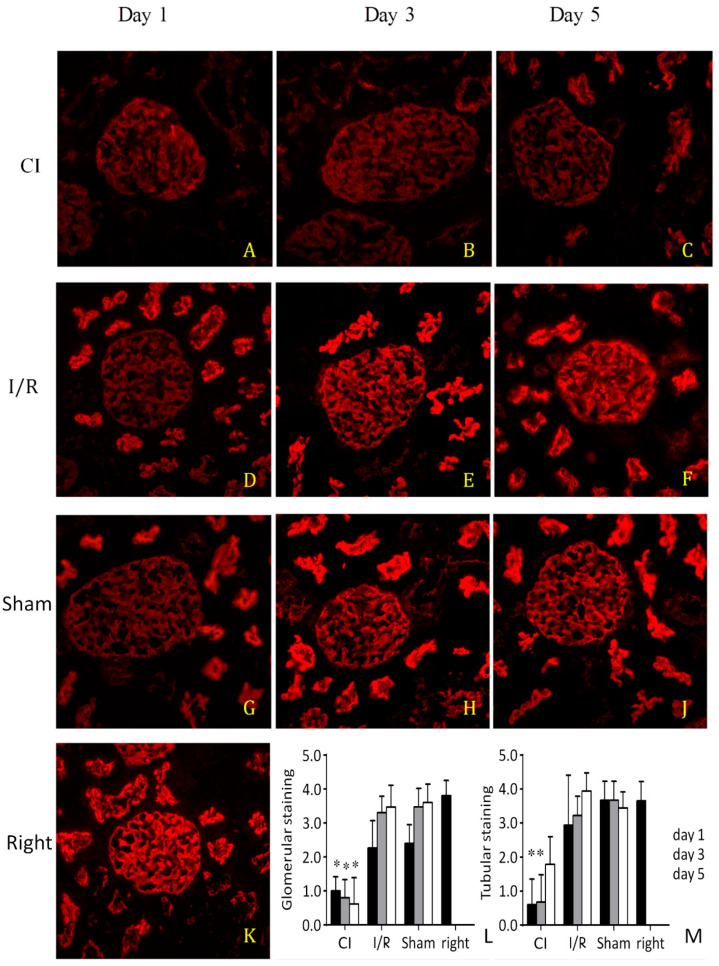
Indirect immunofluorescence for ezrin. Representative images of operated left kidneys, one (**A**,**D**,**G**), three (**B**,**E**,**H**), and five (**C**,**F**,**J**) days after surgery. First row (**A**,**B**,**C**): cold ischemia (CI). Second row (**D**,**E**,**F**): ischemia-reperfusion (I/R). Third row (**G**,**H**,**J**): sham. Fourth row: unoperated right kidney from a sham animal (**K**), (400×). Quantitation of glomerular (**L**) and tubular (**M**) ezrin staining intensity (score: 0–4, 0: no, 1: faint, 4: intense, sharp staining). *: *p* < 0.05 vs. corresponding sham (n = 8 in all groups).

Immunohistochemical staining for ezrin demonstrated a homogenous strong expression in control unoperated right kidneys (Figure 4K) and in sham left kidneys 3 and 5 days after surgery (Figure 4H,J). Normally strong staining was reduced proportionally to tissue damage. Staining was significantly weaker in cold-saline flushed ischemic kidneys, demonstrating that ezrin was severely reduced due to flushing with cold saline (Figure 4A–C). Staining of ezrin started to recover first on day 5 in cold-saline flushed ischemic kidneys (Figure 4C). In I/R kidneys, staining of ezrin was similar to control kidneys (Figure 4D–F).

**Figure 5:**
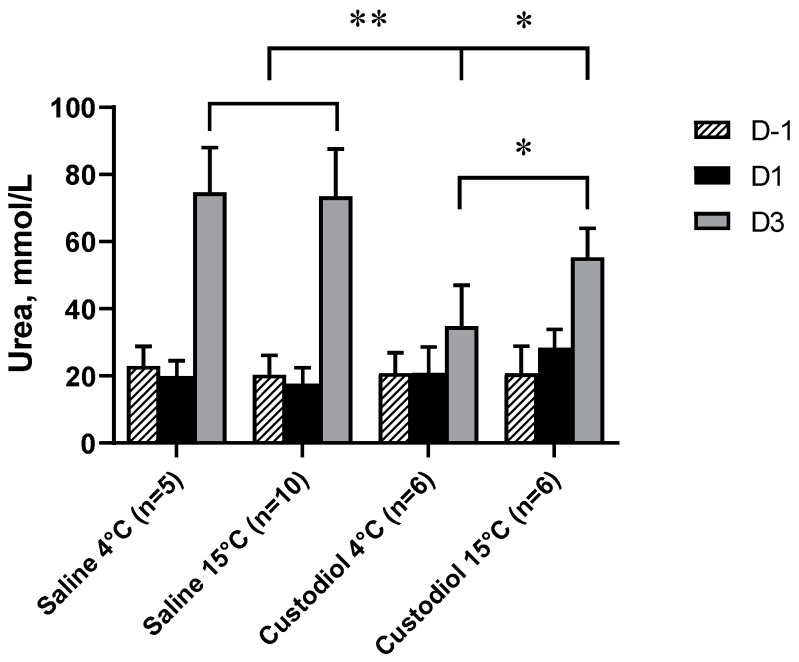
Plasma urea retention on days −1, 1 and 3 (D−1, D1, D3) in rats after flushing the kidneys with saline at 4 (n = 5) and 15 °C (n = 8) or Custodiol at 4 (n = 6) and 15 °C (n = 6) before a 30 min ischemia. Two-way repeated measure ANOVA with Tukey’s post hoc test. *: *p* < 0.05 between Custodiol at 4 and 15 °C and also between Custodiol at 15 °C and saline at both temperatures; **: *p* < 0.01 between saline at 4 and 15 °C and Custodiol at 4 °C.

In order to reveal if the renal injury was caused by flushing with saline or by cold temperature, an additional set of experiments were performed. The kidneys were flushed with saline or Custodiol, a frequently used preservation solution, both at 4 and 15 °C. Plasma urea retention clearly demonstrated that perfusion with saline is harmful, as plasma urea considerably increased at both temperatures (Figure 5). On the other hand, Custodiol caused less injury, and more importantly, the effect of flushing the kidney with Custodiol was more beneficial at 4 than at 15 °C. These results demonstrate that flushing the kidneys with preservation solution at low temperature is favorable and saline is harmful independently from its temperature, even in the case of short preservation times.

## 4. Discussion

In the present study, we demonstrated that flushing the kidneys with ice cold saline before ischemia was associated with significant reduction in renal function compared to non-flushed ischemic kidneys. Tolerated periods of cold ischemia vary between organs but usually do not exceed 24 h of cold storage [1]. However, our results demonstrate that even a short period (30 min) of cold perfusion with saline could be deleterious in living donor transplantation settings.

Cooling donor organs for transplantation to reduce metabolism and ATP loss [37] enables longer donor organ procedures than the previously applied warm blood perfusion [38]. However, organ damage occurs during the cold preservation phase as well. Cold injury has been demonstrated in anoxic limbs: preservation <5 °C was more deleterious than intermediate (10 ± 5 °C) hypothermia [6]. Thus, the development of cold perfusion solutions initiated by Belzer and Collins [3,16] are still ongoing today [39,40]. The anoxic donor tissue with its anaerobic metabolism can generate much less ATP [1,41] and consequent inhibition of the Na^+^/K^+^-ATPase leads to membrane depolarization and eventually cell death, which can be significantly suppressed by reducing metabolic rate of the cells by cooling.

An important mechanism of cold injury is cytoskeletal disruption, probably due to cold anoxia-induced lipid bilayer and Na^+^/K^+^-ATPase dysfunction and consequent cell edema [3,4]. In our study, renal dysfunction due to flushing with cold saline before anoxia was associated with a significant and prolonged loss of ezrin in the kidney. Supporting the role of ezrin in injury caused by cold perfusion even with a preservation solution, ezrin loss and associated apoptosis were recently demonstrated in rabbit kidneys after 4 h of cooling followed by 35 min of warm ischemia [42]. A proteomic analysis of rat renal allografts demonstrated that ezrin was 2.5-fold downregulated in allografts vs. isografts, supporting a role for ezrin loss in allograft damage [43]. Cytoskeletal disruption by flushing with cold saline may be deleterious to microcirculation and parenchyma, and thus, flushing with 4 °C saline is not the optimal preservation approach even in the case of short cold ischemia. We observed the most unanimous and strongest effects on ezrin expression in the glomeruli on day 1 (even some tendency in sham operated kidneys), which was absent in the tubuli and disappeared on consecutive days (Figure 4) in the non-cold-perfused groups. This may explain why ezrin correlated significantly with renal excretory function only on day 1 in the cold-saline perfused animals.

Here, we demonstrated in a separate experiment that saline is harmful to the kidney independently of its temperature as flushing with ice-cold saline before I/R injury induced a large increase in plasma urea. On the other hand, flushing with preservation solution protected the kidney as the urea increase was smaller. In addition, experiments using the preservation solution for flushing the kidney proved that cold temperature (4 °C) is more beneficial than a warmer temperature (15 °C) as the best results were obtained when kidneys were flushed with preservation solution at 4 °C. The design of separate experiments concentrated on the effects of saline and temperature, and therefore we included day −1 to get a healthy baseline urea value and omitted day 5, which shows recovery. Based on these results, we intend to call attention to the fact that flushing the kidney with saline is a wrong approach that introduces a confounding factor in animal experiments and likely decreases the success of living donor transplantations. Our data do suggest that even a short (30 min) perfusion with saline can significantly impair initial graft function. The main problem is that flushing with saline is also in clinical use in the case of living donors and is a generally applied technique in rat transplantation models.

Recent literature data provide strong evidence for the deleterious effects of cold saline perfusion on kidney grafts [39,40]. Even a short (4 h) cold storage period exacerbated mitochondrial dysfunction in transplanted rat kidney autografts [39,40].

The limitations of the study are: 1. No full transplantation procedure was performed in order to specifically analyze the effects of flushing the kidney with cold saline, so the results can be different to some extent in transplantation settings when additional factors influence the outcomes. 2. The study was performed on male rats only so the results may not be extended to the female gender especially because female rat kidneys are more resistant to various injuries. Our study confirms previous observations, indicating that 30 min ischemia is a mild insult in the ischemia/reperfusion rat model [8,9,44]. Thus, the injury and maintenance periods are short, and the repair phase [45,46] starts already on day 3. Flushing with saline either at low or intermediate temperature, however, exacerbated the ischemic injury and delayed the onset of the repair phase.

## 5. Conclusions

Flushing with cold saline may substantially aggravate ischemic damage to renal glomerular and tubular cells. Severe reduction in glomerular capillary function is accompanied by loss of ezrin—an important cytoskeletal protein. Our study draws attention to the potentially adverse effects of perfusion with cold saline and cytoskeletal disruption on the microcirculation and parenchyma in organ preservation even in the case of short cold ischemia. However, such deleterious effects of perfusion with ice-cold saline can be largely prevented by the use of balanced perfusion solution.

## Data Availability

The data presented in this study are available on request from the corresponding author.

## Abbreviations

CI	cold-saline flushed ischemic group
ANOVA	analysis of variance
ATN	acute tubular necrosis
HE	hematoxylin–eosin
I/R	ischemia reperfusion
PAS	periodic-acid–Schiff
PDI	protein disulfide isomerase
SD	standard deviation
SHAM	sham-operated group
TEC	tubular epithelial cell
